# Instigation of indigenous thermophilic bacterial consortia for enhanced oil recovery from high temperature oil reservoirs

**DOI:** 10.1371/journal.pone.0229889

**Published:** 2020-05-12

**Authors:** Neha Sharma, Meeta Lavania, Vipin Kukreti, Banwari Lal

**Affiliations:** 1 Microbial Biotechnology Division, The Energy & Resources Institute, New Delhi, India; 2 Institute of Reservoir Studies Oil and Natural Gas Corporation Limited, Ahmedabad, Gujarat, India; Tallinn University of Technology, ESTONIA

## Abstract

The purpose of the study involves the development of an anaerobic, thermophilic microbial consortium TERIK from the high temperature reservoir of Gujarat for enhance oil recovery. To isolate indigenous microbial consortia, anaerobic baltch media were prepared and inoculated with the formation water; incubated at 65°C for 10 days. Further, the microbial metabolites were analyzed by gas chromatography, FTIR and surface tension. The efficiency of isolated consortia towards enhancing oil recovery was analyzed through core flood assay. The novelty of studied consortia was that, it produces biomass (600 mg/l), bio-surfactant (325 mg/l), and volatile fatty acids (250 mg/l) at 65°C in the span of 10 days, that are adequate to alter the surface tension (70 to 34 mNm ^-1^) and sweep efficiency of zones facilitating the displacement of oil. TERIK was identified as *Clostridium* sp. The FTIR spectra of biosurfactant indicate the presence of N-H stretch, amides and polysaccharide. A core flooding assay was designed to explore the potential of TERIK towards enhancing oil recovery. The results showed an effective reduction in permeability at residual oil saturation from 2.14 ± 0.1 to 1.39 ± 0.05 mD and 19% incremental oil recovery.

## Introduction

Various methods have been employed in oil recovery that comprises of: primary phase, which utilizes natural energy forces of reservoir for oil recovery, secondary phase includes the injection of water that pushes the oil towards the production well. After this recovery, remaining crude oil in the wells makes up to two-third of the total oil reserves. The present method of oil recovery is based on surfactant flooding, polymer flooding, alkaline flooding, and injection of steam (reduces the viscosity of oil and facilitating the recovery of oil) [1, 2]. Chemical and thermal methods are not economically feasible and therefore may affect the reservoir environment [3].

Current global energy production through oil is about 80–90%. During the primary process of oil recovery, between 30 and 40% of oil can be contributed by primary oil recovery, while additional 15–25% can be recovered by secondary methods. Around 35–55% of residual oil is left behind in the reservoir [4]. The alternative approach is developed to enhance the recovery of oil includes either injection of micro-organism or their products which serve as a cost effective solution. The microbial biomass has a tendency to clog the permeable zones of reservoirs. The microbial cell will grow at the pore throats restricting the flow of water, further it will divert the water path and displacing the unswept oil from its position this process is known as bacterial profile modification. The microbial metabolites such as gases, biosurfactants or bio-emulsifiers, biopolymers, solvents, acids affect the overall sweep efficiency, reduces the viscosity of crude oil and surface or interfacial tension by accumulating at the interface of immiscible fluids. There are various microbial activities (hydrocarbon metabolism) based methods which improves the recovery of residual oil from reservoirs [3, 5, 6].

The biosurfactant are amphipathic molecules with hydrophobic as well as hydrophilic groups, which impart functional properties. They are high value surface-active products due to their specific activity, low toxicity, ease of application and biodegradability. Biosurfactant are of two types, low molecular weight biosurfactant (glycolipids; lipopeptides) and high molecular weight biosurfactant (lipopolysaccharides, lipoproteins or a combination of these). The biosurfactant are diverse groups of the molecule that reduces the overall surface and interfacial tension between the hydrocarbon and aqueous phases which further mobilize the entrapped oil [7, 8].

Microbial Enhanced Oil Recovery (MEOR) is an environmentally friendly method posing several advantages over the other recovery processes (chemical, thermal, polymer, and surfactant flooding). MEOR is an affordable process as it involves fermentation of low cost substrate or raw material for the production of useful microbial products (biosurfactants, gases and volatile fatty acids) [9].The present investigation is focused on evaluation of indigenous consortia towards the enhance oil recovery. The study includes the determination of physicochemical analysis of formation water and oil, development and characterization of indigenous bacterial consortia for the production of metabolites (biosurfactant, volatile fatty acids). The potential of indigenous consortia was analyzed through core flood assay in which gradual reduction was monitored in the permeability of the core due to the plugging induced by microbes. The consortium (TERIK) was appeared to be nontoxic and environmentally attractive option for the field trials.

## Materials and methods

### Sampling site

The oil reservoir is owned by Oil and Natural Gas Corporation (ONGC) Limited, Gujarat with the stretch of 30 km. The average annual rainfall is around 723 mm. The formation water was accrued from Gujarat reservoir named TERIK (Kalol) with the bottom hole temperature of 60–75˚C during the month of July 2015. The sample was inoculated onshore into pre-sterilized anaerobic media vials. The sample was transported to The Energy & Resources Institute (TERI) for further investigation.

### Analysis of formation water and oil

The formation water was analysed for the presence of hydrogen ion concentration (pH), elements, anions (Chloride, Sulfate, and Fluoride) and heavy metals. The analysis was done by the API (American Petroleum Institute) and APHA (American Public Health Association) standard guidelines. Heavy metals were analysed in formation water, including arsenic, chromium, silver, cadmium, zinc, nickel and copper. Salinity was performed using AgNO_3_ titration method. The total petroleum hydrocarbons (TPH) were analysed as described by Lavania et al., (2015) [10].

### Enrichment and screening of indigenous consortia (TERIK) for enhanced oil recovery

An enrichment technique was employed in isolation of indigenous microbial consortia (TERIK) for enhanced oil recovery from the formation water sample. Around 10% v/v formation water sample was inoculated in Baltch media. The composition of Baltch medium (g/l): 2g of NH_4_Cl, 0.2g of MgSO_4_.7H_2_O, 2.6 g of NaHCO_3_, 10g of Molasses, and 1g of NaCl. To maintain the anaerobic condition, oxygen indicator and reducing agent was added in 1 liter of medium; further sparged with nitrogen gas to remove the dissolved oxygen. The bottles were covered with aluminum crimp and autoclaved for 15 min at 121°C. The autoclaved media were inoculated with 3 ml of formation water under the aseptic conditions and incubated at 65°C for 10 days. All the experiments were performed in triplicates in 67 ml anaerobic Wheaton serum bottle. After 10 days of incubation, the bottles were analyzed for various metabolites as described in quantification section.

### Characterization of TERIK

For identification of bacterial consortia TERIK, total genomic DNA was extracted and amplification was performed using universal primers (27f and 1492R). The PCR reaction was purified using the QIAGEN gel elution kit and cloned through the PGEM-T kit as described by Sharma et al., 2018 [11]. The phylogenetic tree was constructed using the neighbor-joining method in MEGA version 6.06 packages [12]. The tree topologies were estimated with 1000 bootstrap data sets. The sequences were submitted in the NCBI GenBank database. The morphology of TERIK was studied by scanning electron microscopy (Zeiss EVO MA 10). Under aseptic condition, sample was immersed for 2 to 4 hours in 2.5% of glutaraldehyde. Followed by washing with 0.1M phosphate buffer and dehydrated with ethanol solution. The sample was air dried and coated with thin layer of metal (gold and palladium).

### Effect of carbon and nitrogen source on production of metabolites

The effect of various carbon sources on anaerobic growth of TERIK was assessed by growing the consortia in Baltch media supplemented with 1% of glucose, glycerol, molasses and fructose. Similarly, the effects of various nitrogen sources on growth were also determined by growing the consortia in the medium supplemented with 0.2% (w/v) yeast extract, 0.2% (w/v) of Urea, 0.2% (w/v) of NH_4_Cl and 0.2% (w/v) NH_4_NO_3_. The effect of carbon and nitrogen sources on anaerobic growth and biosurfactant production of TERIK was monitored [13].

### Quantification of microbial growth and metabolites

The microbial growth was monitored spectrophotometrically at 600nm. Further, growth of micro-organism was estimated through biomass, volatile fatty acids and biosurfactant. The biomass was measured by centrifugation of media (centrifuge 5810 R) at 10,000 rpm for 10 minutes at 4°C. The pellet was dried at 37°C and weighed until the stable weight was obtained. The volatile fatty acids were analysed by GC -7890A (Agilent Ltd. USA) facilitated with flame ionized detector and DB-WAX etr column (30m × 530μm × 1μm). The calibration curve was prepared with the standards and the R^2^ value was 0.998 [14]. The bio-surfactant was extracted by solvent method; the cell supernatant was acidified with 6N HCl until pH reached at 2.0. The precipitated biosurfactant was centrifuged for 10 mins at 10000 rpm and extracted using equal volumes of chloroform: methanol mixture (2:1). The mixture was kept undisturbed for several minutes in order to facilitate phase separation. The organic phase was collected and the crude biosurfactant was dried under vacuum at 45°C [15].

The cell free supernatant was analyzed for surface tension with CSC DuNouy Tensiometer (Cole Parmer India) equipped with a platinum ring. The un-inoculated broth, serve as a control, was used and the surface tension was calculated according to the following formula:

λ=(r×h×d×g)/2 (1)

Where, λ = surface tension (mN/m); d = density (g/ml); g = gravity (980 cm/s2); r = capillary radius (0.025 cm); h = height of the liquid column (cm) [10]. Molish test was performed to detect the presence of carbohydrate in biosurfactant sample [16]. The biosurfactant was also tested for oil spreading and drop collapse method. The functional group of biosurfactant was determined by Fourier transform infrared spectroscopy (ATR-FTIR model Nicolet, 6700) equipped with an ATR accessory [17, 18].

### Toxicology studies

The study was conducted to establish the toxicity of “TERIK” administered by the oral route to the mice (*Mus musculus*). A 1.0 ml of the test material containing around 1 x 10^6^ CFU was administered orally to mice (6 male and 6 female). They were assigned to the dose groups, control and test. All the animals were observed at least once during the first 30 minutes after dosing, and periodically for 21 days. The body weight, organ weight (Adrenals, Heart, Kidneys, Spleen and Lungs) and mortality was recorded. The hematological parameters, glucose, BUN (blood urine nitrogen), SGPT (Serum Glutamate Pyruvate Transaminase), total proteins and albumin were studied for 21 days of the investigation [19]. The study was performed by the National Toxicology Centre (APT testing & Research PVT.LTD), Pune.

### Ethics statement

Toxicological study of TERIK was performed in mice by APT Testing And Research Private Limited, Maharashtra, India under EPA 712-C-96-322, OPPTS 885.3550 guidelines, adopted in February 1996. The study was reviewed with the OECD principles of Good Laboratory Practices & ISO 17025.

### Core flood assay

The core flood experiment (an experiment under simulated oil reservoir conditions) was performed at IRS, ONGC, Ahmedabad to determine the water cut and selective plugging of permeable zones of the reservoir with isolated consortia TERIK. The characteristic conditions used in the study were described in Table 1. The core was saturated with the formation water and crude oil. The original oil in place was around 12.6 ml. The formation water was flooded till the point; no oil was coming out of the core that shows the pore volume of the core which was around 21.1ml. Subsequently, TERIK consortia (1 x 10^6^ CFU) along with the media were injected and the apparatus was incubated at 65°C for 10 days. Differential pressure was determined along with the monitoring of end point permeability to evaluate the efficiency of consortia towards the selective plugging of core.

**Table 1:** Petro-physical parameters in core flood study.

Characteristics	Value
***Core flood study***	
Length (cm)	10.11
Diameter(cm)	3.87
Formation water/ crude oil	Kalol#35
Pore volume (cc)	21.1±0.5
Porosity (%)	17.8
Water saturation, Swi,%PV	40.1±1
Original oil in place(OOIP),(ml)	12.6±0.4
Irreducible oil saturation,Sor,%PV	40.4±0.8
Water permeability at residual oil,Kw@sor (mD)	2.14±0.1
Water permeability at residual oil,Kw@sor (mD)after treatment	1.39±0.05
***Operational conditions***	
Temperature	65°C
Pressure, psi	1300
Microbial culture	TERIK
Rate of injection of fluid(cc/min)	0.1

The percentage of oil recovery was measured using following equations:

OilrecoveryefficiencyORE(%)=(Totalvolumeofoilrecovery÷Originaloilinplace)×100 (2)

Therefore, enhanced oil recovery (EOR) was estimated by,

EOR(%)=OREm−OREw (3)

Where, OREw stands for oil recovery efficiency at the end of subsequent water flooding; OREm is an oil recovery at the end of bacterial injection [14].

The treated core was further visualized through a scanning electron microscope (10KV in Zeiss EVO MA 10) in order to determine the interaction between the microbes and the pores of the core. Micrograph of the core was captured digitally using digital image transfer recognition program Zeiss.

### Statistical analysis

All experiments were performed in triplicates. One-way analysis of variance (ANOVA) was used to detect the significant differences among the variables. The probability (p) <0.05 represent the significant difference.

## Results

The present study focused on implementing Microbial Enhanced Oil Recovery (MEOR) technique in high temperature oil reservoirs. As a consequence of long time exploitation with synthetic compounds, fracturing and acidizing liquids has affect the overall composition of formation water and furthermore affect the types and number of microorganisms in the oil wells.

Pointing toward an effective methodology for potential residual oil recovery, attributes of the formation water/oil was studied as tabulated in Table 2. The formation water was slightly alkaline with the pH of 7.83±0.2. The presence of heavy metals, toxic anions was also monitored. There were no traces of heavy metals (Arsenic, Cadmium, Chromium, Copper, Zinc, Silver and Nickel) in the sample or they may be below the detection limit. The CHNS of formation water plays an important role in the secondary metabolite production such as volatile fatty acids, biosurfactant [20]. The total petroleum hydrocarbon of the oil was estimated with the following composition: 56% aliphatic, 26% aromatic and 18% NSO and other compounds.

**Table 2:** Physico-chemical parameters of formation water.

Physicochemical parameters	Test method	Results
*pH*		7.83±0.2
*Salinity(mg/l)*	Titration	1612±25
*Heavy Metals(mg/l)*		
Arsenic	IS3025 PT 37:1988	ND
Cadmium	APHA3100(B)	ND
Chromium	APHA3500(B)	ND
Copper	APHA3111(B)	ND
Zinc	APHA3100(B)	ND
Silver	APHA3113(B)	ND
Nickel	APHA3111(B)	ND
*Anions (mg/l)*		
Chloride	IS3025 Pt 32: 1988	1065±15
Fluoride	APHA 4500 (F-D)	0.4±0.01
Sulphate	IS 3025 Pt 24: 1986	65±1
*Elements (ppm)*		
Carbon	APHA/IS:1350	910±30
Hydrogen	APHA/IS:1350	54±1.5
Nitrogen	APHA/IS:1350	46±1
Sulphur	APHA/IS:1350	15±0.2

ND means Not Detected

### Enrichment of indigenous thermophilic consortia TERIK for enhanced oil recovery

To cultivate the indigenous consortia TERIK for enhanced oil recovery, formation water was injected into Baltch media and was incubated at 65°C. The microbes indigenous to the oil reservoirs seem to be the best choice as it acclimatized to the harsh reservoir conditions. The indigenous microbes are capable of producing other secondary metabolites that facilitate enhanced oil recovery by reducing the interfacial tension between the aqueous phase and hydrocarbon [21].

The enriched anaerobic microbial community was analyzed for biomass, which was around 600 mg/l and the absorbance was 0.3 at 600nm. The extracted bio-surfactant was 325 mg/l along with 250 mg/l of volatile fatty acids were estimated at 10 days. The volatile fatty acids and gases produced by micro-organism plays significant role in the mobilization of crude oil.

The efficiency of bio-surfactant produced by consortia TERIK was determined by estimating its impact on the surface tension of the media. The surface tension was reduced from 70 to 34 mNm^−1^ after 10 days of incubation, which demonstrated 51.4% of overall reduction. Biosurfactant are amphiphilic molecules which reduce the surface tension between oil-water/oil-rock interfaces and furthermore alter the wettability of the reservoir which leads to the mobility of trapped oil. The biosurfactant produced in this study further supports the enhanced oil recovery by giving positive oil spreading and drop collapse test (S1-S3 Figs in S1 File). According to Maneerat et al., 2007, they had screened emulsification activity of biosurfactant produced by various bacterial strains and also monitored the efficiency of biosurfactant through zone clearance method [22].

**Fig 1:**
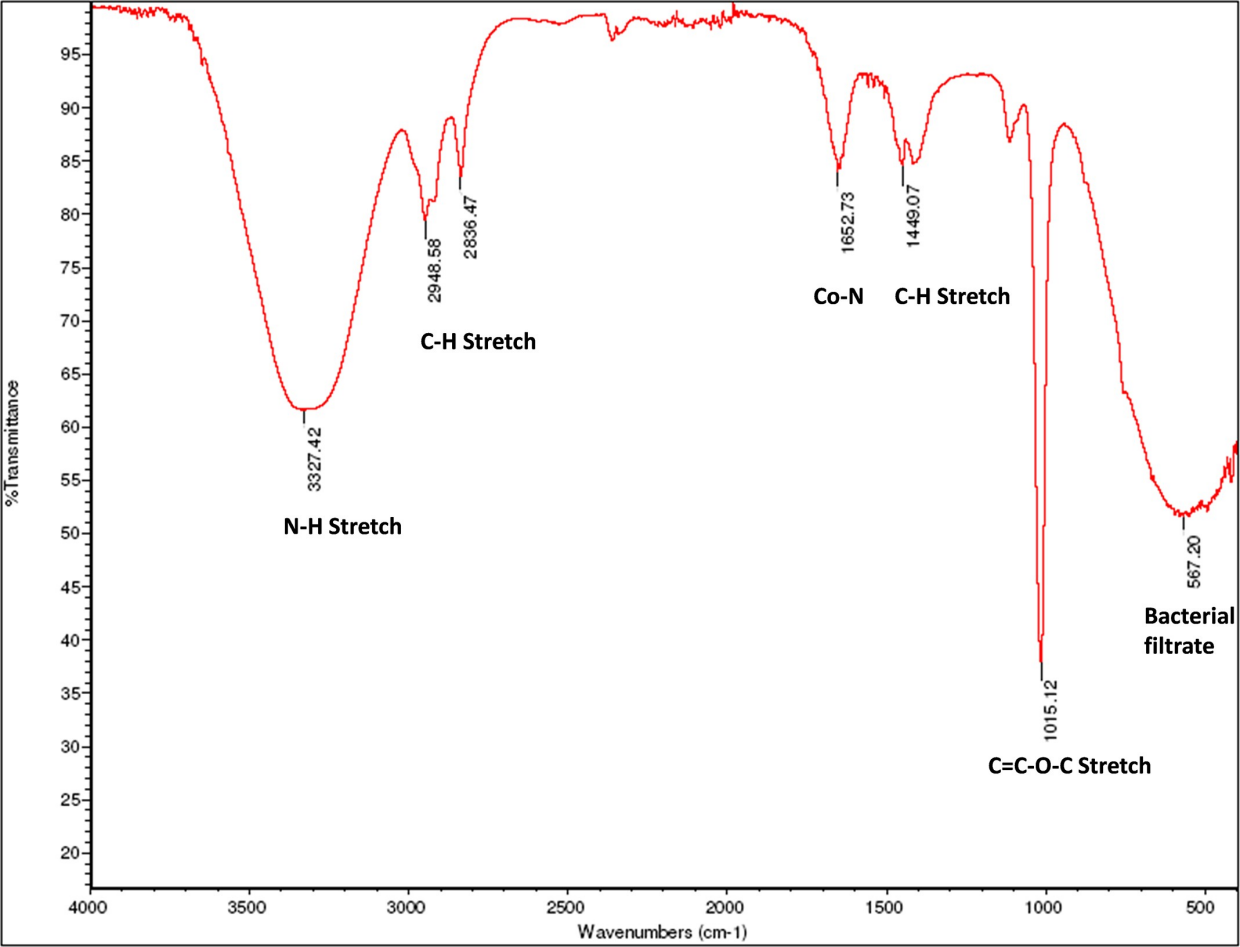
FTIR spectra of the biosurfactants produced by TERIK.

The functional groups present in the bio-surfactant produced by TERIK were analyzed by FTIR spectroscopy. The infrared spectrum representing intense band at a wave number 3327 cm^-1^ showing stretching mode of the N-H bond; wave number 2948 to 2836 cm^-1^ and 1449 cm^-1^ indicating C-H stretching that defines the presence of aliphatic chains. The characteristic bond for the presence of amide I and amide II was shown by wave number 1652 cm^-1^. The strong peak at 1015 cm^-1^ represent the C = C-O-C, C-O, C-O-P, P-O-P vibrations of polysaccharide. The wave number 567 cm^-1^ shows protein vibration present in bacterial filtrate as depicted in Fig 1 [23, 24].

### Identification and characterization of TERIK

**Fig 2:**
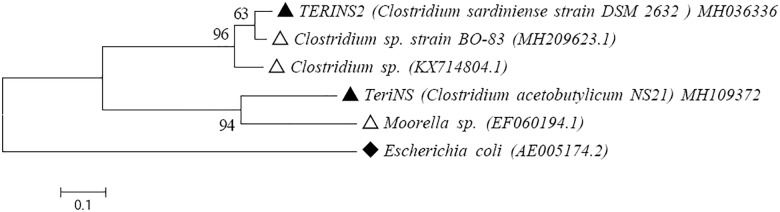
A phylogenetic tree based on 16S rRNA indicating the isolated strain with closely related members. Topology was constructed using Neighbor Joining method in MEGA 6.06software. Accession number obtained from NCBI database (MH036336 and MH109372). Bootstrap value (n = 1000 replicates) of >75% are reported. Scale bar = nucleotide substitution per site. Out-group species were indicated with Δ and ♦.

TERIK consortia were characterized on the basis of 16S rRNA sequencing. The sequences of consortia TERIK were analyzed using BLAST. The consortia showed the presence of genus *Clostridium* species with the accession number of MH036336 and MH109372. The phylogenetic tree was constructed with the closely related match through MEGA 6.06 software (Fig 2). *Clostridium* species were the most commonly used microorganisms in all field trials. Few reports represent the presence of *Bacillus atrophaeus* in the environment sample [25]. Researchers investigated the distribution of microbial communities such as Firmicutes, Bacteroidetes, Actinobacteria, Thermotogae in the oil fields of China [26]. Various studies were reported on injection of mixed anaerobic or facultative anaerobic bacteria (*Clostridium*, *Bacillus*, *Pseudomonas*, *Arthrobacterium*, *Micrococcus*, *Peptococcus*, *Mycobacterium* etc.) in case of field trials; these microbes were selected on the basis of their ability to produce gases, acids, solvents, polymers, surfactants, and cell biomass [27].

The *Clostridium* species have been applied in MEOR studies due to their ability to produce various metabolites which play important role in microbial enhanced oil recovery process [28]. A previous report showed that the pure strain of *C*. *tyrobutyricum* (DSMZ 663) has the capability to produce gas at a salinity of 100 g/l by utilizing molasses as carbon source. The composition of the gas was about 83% carbon dioxide, and 15% hydrogen [29].

**Fig 3:**
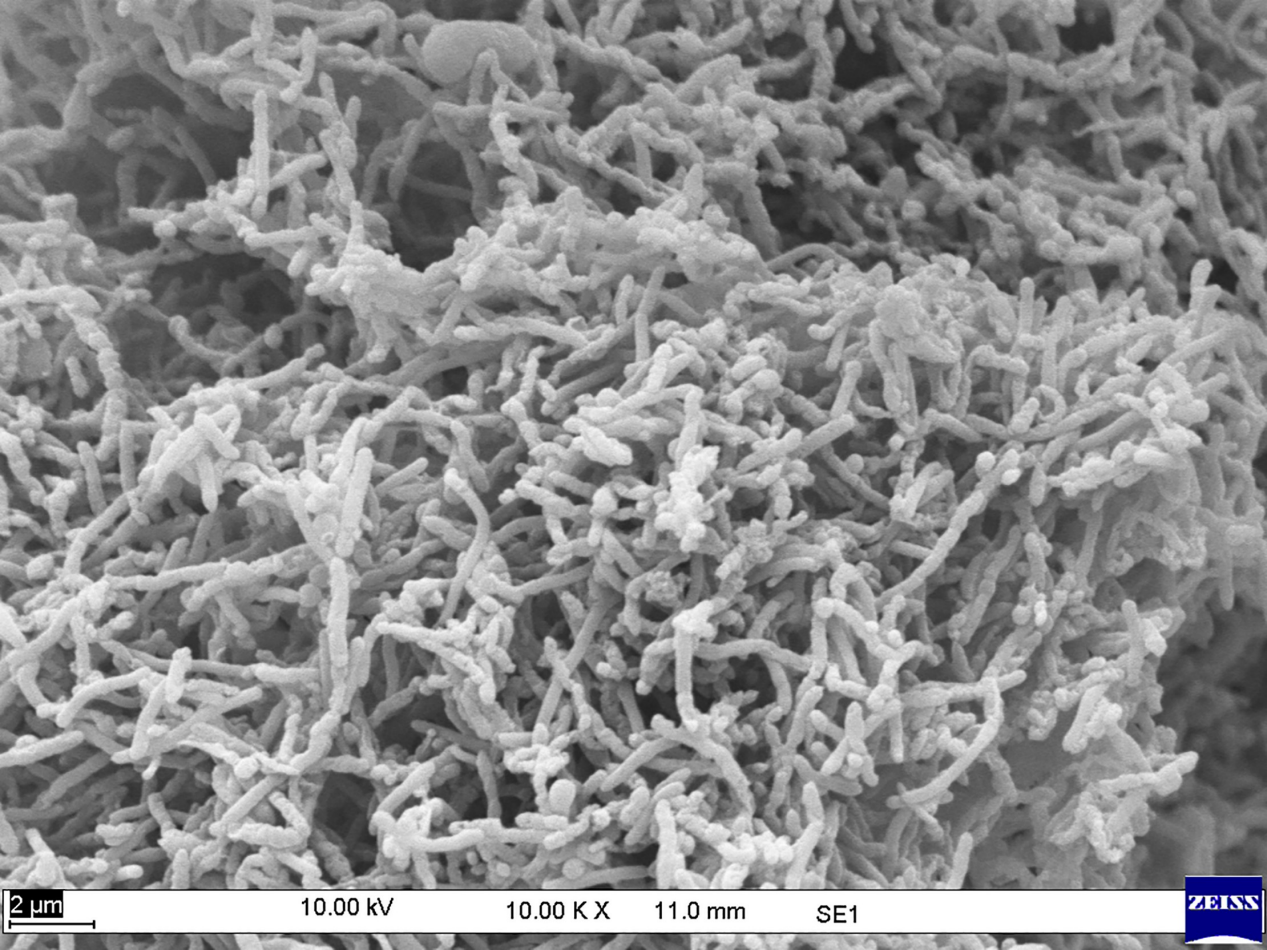
Morphological characterization of TERIK through scanning electron microscopy at 10KX magnification, bar 2μm.

The morphology of consortia TERIK was analyzed through scanning electron microscopy. The scanning electron micrographs revealed the presence of a mesh of rod shaped bacteria (Fig 3).

### Optimization of carbon and nitrogen sources

**Fig 4:**
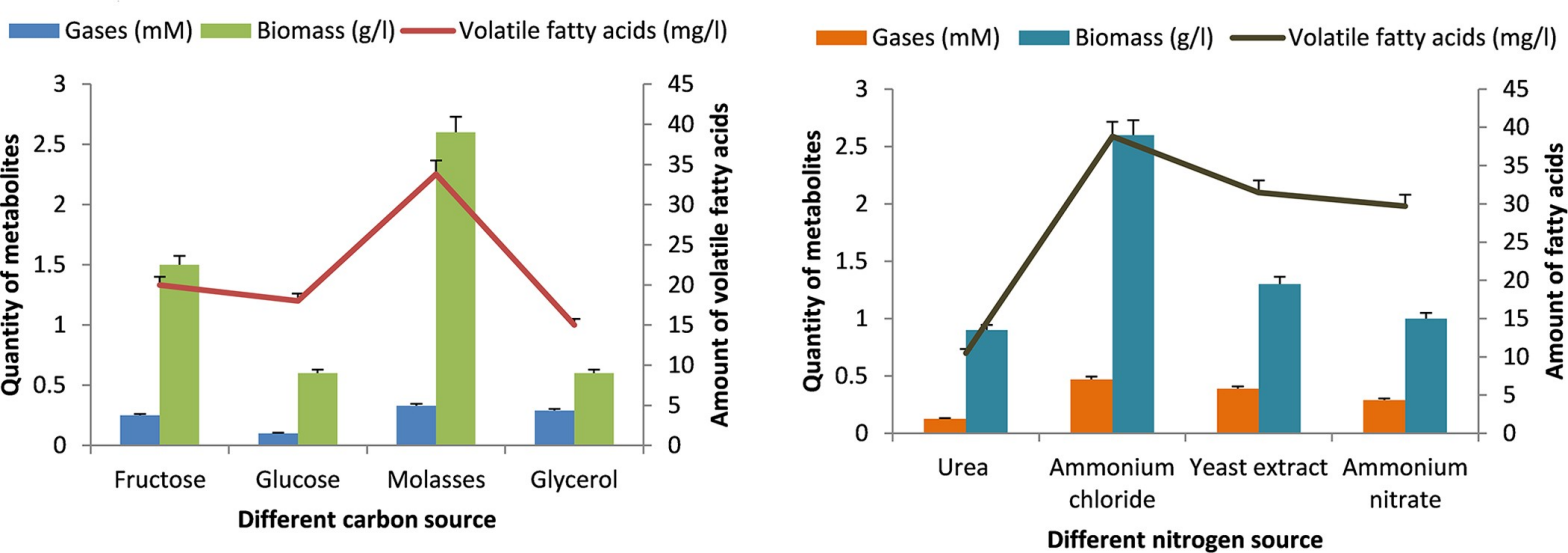
Represents the effect of various media components on the growth pattern of consortia TERIK. (A) Effect of nitrogen source, (B) Effect of carbon source.

For the present analysis, four different carbon sources was selected out of which molasses was appeared to be significant carbon source with respect to gas, volatile fatty acid and biomass production. Media with molasses showed 33.8 mg/l of volatile fatty acid and 0.33 mM of carbon dioxide. The effect of glucose was slightly inferior over fructose. Minimum growth was observed in case of glycerol. For nitrogen source utilization, ammonium chloride (NH_4_Cl) contributed significantly well towards microbial growth. Yeast extract was also a suitable source for biomass generation. The minimum amount of microbial growth was depicted in a medium with urea as a nitrogen source (Fig 4).

### Pathogenicity assay

In the present study, the acute oral toxicity of indigenous microbial community (TERIK) of oil well was evaluated. The single oral dose of the test material was administered to the mice, which revealed no clinical sign of illness. A total of twelve mice of 6 male and 6 female was tested at one dose level. “TERIK” did not cause any mortality in the male and female mice treated at the dose of 1.0 X 10^6^ CFU. There was no statistically significant decrease or increase in the absolute weights of the organ in the test group when compared with the control group during 21 days of observation period, following dosing. The body weight of mice was gradually increased in the control and test mice sets. The alteration in the body weight may reflect the general health status of animal [30].

The body weight gain was observed in all the animals which suggest that test material did not interfere with the normal body metabolism. The evaluation of blood hematology and biochemistry contribute an insight to potential damage brought by the test material in hepatic and renal functions. An SGPT test was conducted to evaluate any damage to the liver [31]. The slight reduction in ALT and AST levels in the treated mice in both sexes compared to the control set could suggest that test material may not have any hepatotoxic effect and might not have any effect on the heart. Cultured tissues (lungs, spleen, kidney, adrenals and heart) showed no live anaerobic bacteria in test material. The results from the necropsy revealed no abnormalities were observed or detected in the test when compared with the control group animals. A reduction in the total protein content, albumin and globulin is an indication of damaged hepatocellular function [32]. Study of blood urea nitrogen (BUN) level reflects renal problems. The BUN levels of control and test mice suggested the normal renal function in the test animal. Hematological estimation is used to determine the detrimental effect on the blood [33, 34]. The result of present studies showed no effect on RBC’s, WBC’s, PCV and hemoglobin values of treated mice indicates that erythropoiesis, the fragility of RBC and morphology are not affected [35].

In statistical analysis, ANOVA was performed in which p-value (0.01) was less than 0.05 which clearly represent that there was no significant difference in the mean value of the variables (control and test) with the confidence level of 95%. The entire population of experimental model appeared normal and showed no clinical signs of intoxication after dosing till the end of the study.

### Enhanced oil recovery

**Fig 5:**
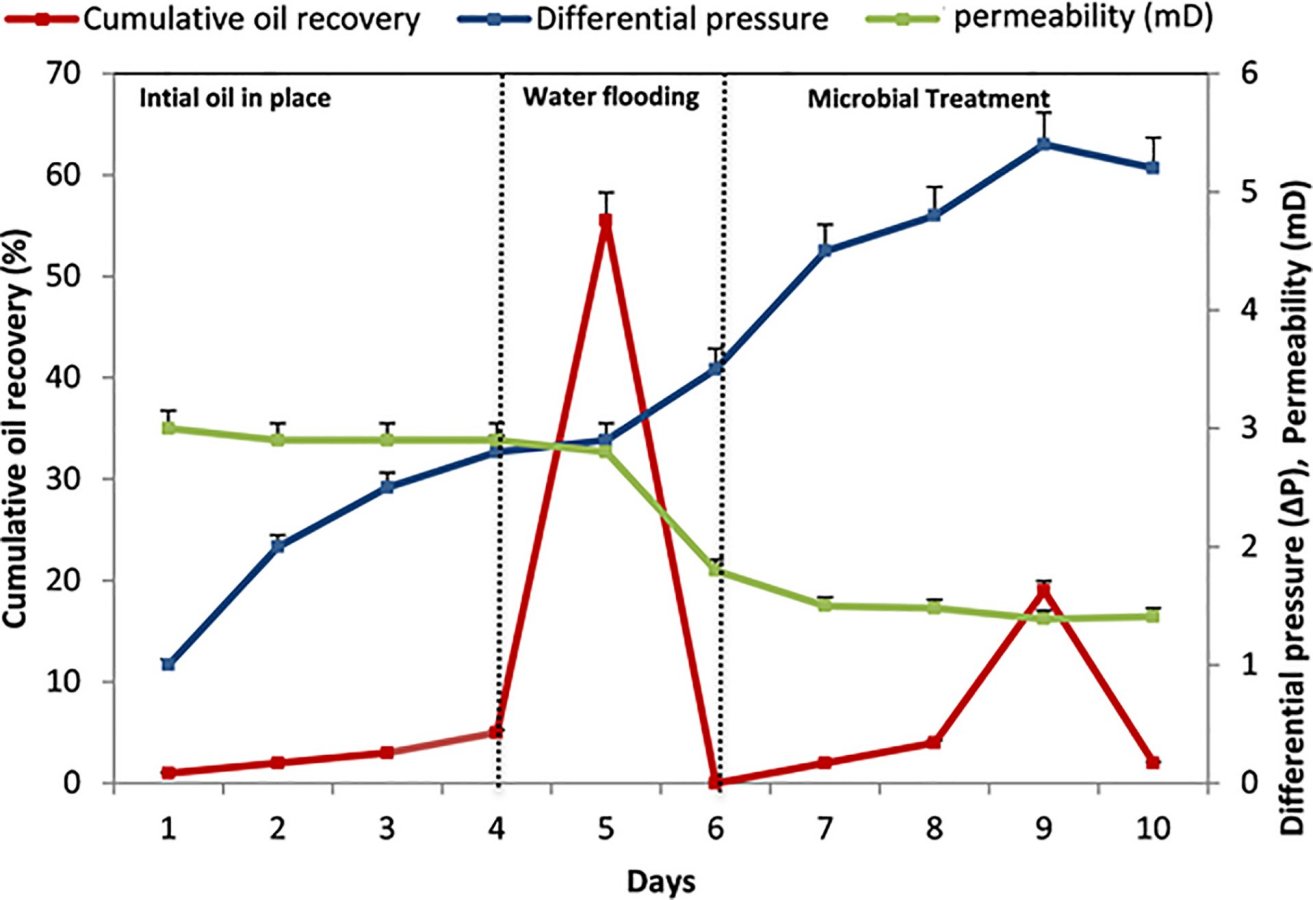
Cumulative oil recovery in core flood assay.

To assess oil recovery a bench scale technique (core flood) was employed. The column was saturated with the oil and microbial solution; water flooding was further performed to determine the percentage of incremental oil recovery [36]. Firstly, the core was flooded with water, which leads to 17.8% of water saturation. Further, it was saturated with the oil that gives 40.4± 0.8% irreducible oil saturation (12.6 ± 0.4 ml). At the MEOR stage, the nutrient media along with TERIK at their exponential phase was injected at a rate of 0.1cc/min. The OOIP was 12.6 ml, Sorwf (oil recovered after water flooding) was 7 ml and Sorbf (oil recovered after biosurfactant flooding) was 2.4 ml (Table 1). The microbial consortia were capable of producing biosurfactant, biomass, which simultaneously facilitating oil recovery. The consortia TERIK was observed at the outlet after 10 days of incubation. In this assay, overall 75% of oil was recovered, out of which 19% of incremental oil was recovered at 65°C due to the microbial action (Fig 5).

The water permeability was also reduced in core flood test from 2.14 ± 0.1 to 1.39 ± 0.05 mD which clearly indicate selective plugging of pore space resulted in a gradual increase in the differential pressure across the core from 3.5 to 5.4 psi. This process improves the sweep efficiency across the core [37]. These results showed the ability of consortia for field implementation. After the core flood assay, core was morphologically characterized through scanning electron microscopy.

**Fig 6:**
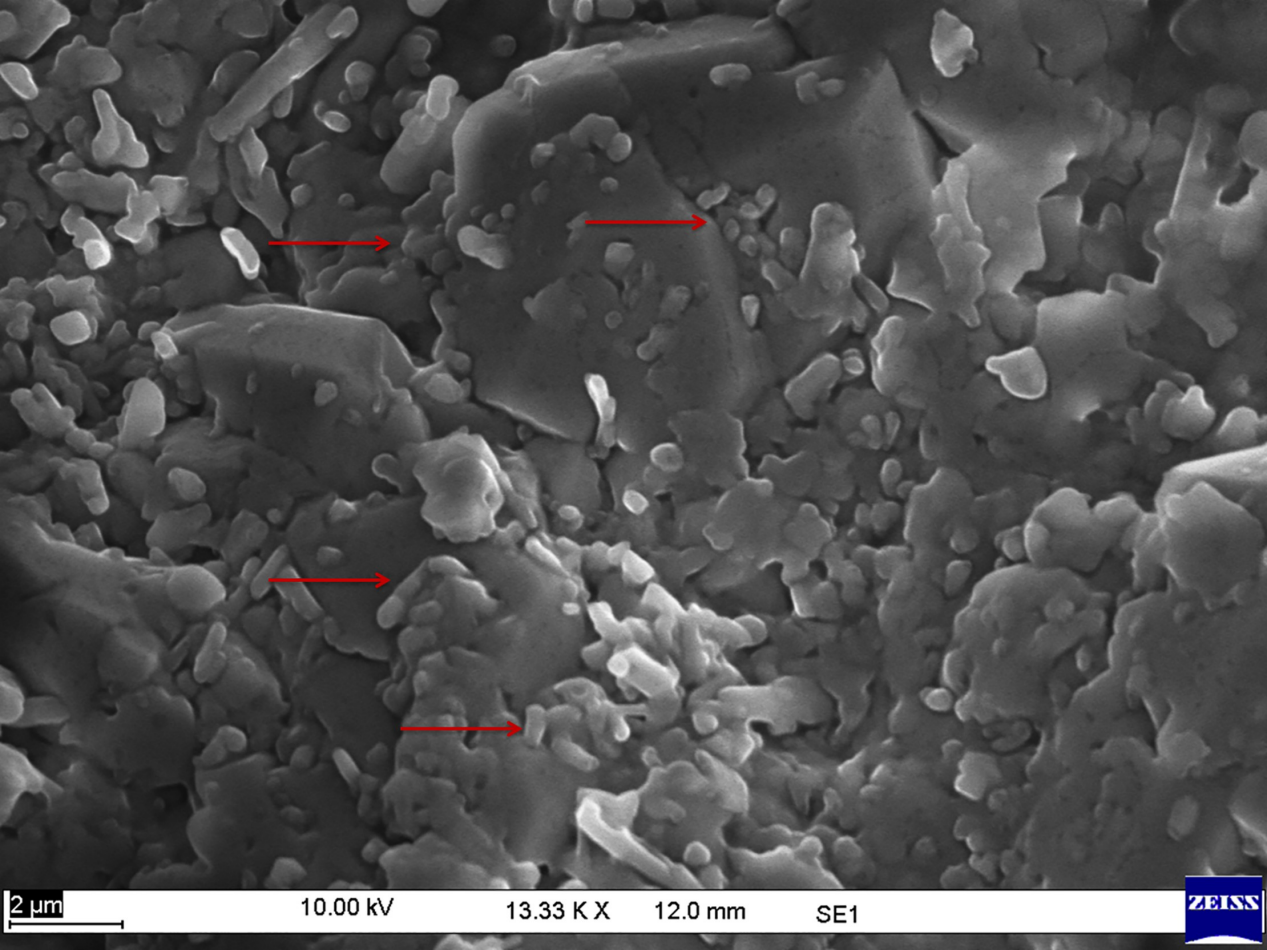
Scanning micrograph of treated core indicating the plugging pattern induced by TERIK where, red arrows illustrated the bacterial growth in core flood assay (scale bar 2 μm).

The micrograph showed the plugging phenomenon within the pores of core. The bacterial cells of TERIK were entirely clogging the core and lead to the incremental oil recovery (Fig 6).

## Discussion

The mechanism of bacterial biomass in MEOR involves the selective plugging of high permeability zones of the reservoir where the microbes grow and forms barriers that restrict the undesirable water flow. This tends to displace un-swept oil and increasing oil recovery [38]. The advantage of microbial permeability profile modification process was that it does not interfere with the normal water flood operation which makes it eco-friendly and cheapest mechanism [39]. The physical and chemical properties of produced water from oil reservoir and oil bearing rock can determine the success of MEOR method [40].

The pressure of the reservoir is not a limiting factor for the growth of consortia. There are a number of reports published on the survival of a microorganism at higher pressure [11].Few reports showed *Clostridium* species can produce 0.0034 moles of acid per kg of molasses that tend to cause dissolution of carbonate rock and hence improved its porosity and permeability [41]. A researcher shows a correlation between bacterial genus *Clostridium* and VFA concentration that suggest the abundance of some archaeal/bacterial genus towards fermentation or Methanogensis [42].

“TERIK” was found to be safe to mice in acute oral toxicity analysis. Thus, becomes a suitable candidate for field implementation (for enhanced oil recovery) due to its non-toxic and non-pathogenic behavior and have no harmful impact on the environment as well as on the health of workers [19].

The field studies in China confirmed the proposed MEOR mechanisms. Numerous field trials showed the gradual increase in the injection pressure soon after bacteria injection. This can be attributed to the plugging of pore throats by the microbes, production of biosurfactant, or gases. At a certain stage the injection pressure began to decline. This is due to the surfactant produced by bacteria that reduces the interfacial tension between oil and water [43]. Researcher have investigated the autochthonous spore former (*Paenibacillus ehimensis* BS1) from oil fields of Oman. The isolated bacterium shows the biotransformation activity and that eventually improves the recovery of oil [44]. An experimental study was conducted that showed the effect of high pressure (10 MPa) and atmospheric pressure on bacterial metabolism. It was observed that high pressure negatively impacted bacterial growth [45].

A researcher Coty et al., (1983) reported a field trial in Arkansas, where *Clostridium acetobutylicum* was injected in fresh water with 2% of crude beet molasses for a period of 6 months. After 70 days of injection, a breakthrough occurred, *Clostridium* fermented molasses into short- chain fatty acids, ethanol, 1-butanol, acetones and CO_2_. The production of oil was augmented from 0.6 bbl/ day to 2.1 bbl/day [46].

Few reports demonstrated the utilization of genetically modified bacteria in microbial enhanced oil recovery, that are capable of producing 11.44 mmol L^-1^ CO_2_ at 45°C and shows 17–25% of oil recovery [47]. A report by Gonzalez et al., (2003) shows the activity of organic acids in core flood experiment where the organic acid lowers the pH that mobilize the oil, and facilitate 5–10% of oil recovery [48]. Another of Jinfeng et al., (2005) represents 5.6% of incremental oil recovery after 7 days of incubation at 70°C [49].

In MEOR process, injection of microbes capable of producing *in situ* biosurfactant in oil reservoirs is relatively cost-effective when compared with the injection of biosurfactants products [50, 51]. The production of biosurfactants in anoxic oil reservoirs is crucial for *in-situ* MEOR applications [52]. Nerurkar et al., (2009) isolated *Bacillus licheniformis* TT33 from hot water spring. Its biosurfactant reduces the surface tension from 72 to 34 mNm^-1^ [53]. In India, joint research of ONGC, IRS, and TERI leads to the improved Microbial technology of a cultured set of microbes that could survive temperatures as high as 90°C. These microbes were effectively tested in the oil wells of Gujarat and Assam. The increment of threefold in oil recovery has been reported [11].

## Conclusion

The isolated consortia TERIK had potential towards the enhanced oil recovery in the high temperature reservoir. TERIK was selected based on its capabilities to produce the substantial amount of biomass and other secondary metabolites (volatile fatty acid, and biosurfactant). Furthermore, the consortium had ability to reduce surface tension from 70 to 34 mN m^-1^. TERIK was characterized to be *Clostridium* sp. The oil recovery efficiency of TERIK was tested in a core flood assay in which produced biomass interacted with the porous medium of the core that brings about the plugging and facilitating around 19% of enhanced oil recovery. This study confirms the importance of TERIK for oil industries.

## Supporting information

S1 File (DOCX)

S1 Graphical abstract (PNG)
