# Supplementary material for: Instigation of indigenous thermophilic bacterial consortia for enhanced oil recovery from high temperature oil reservoirs
Source: PLoS One. 2020 May 12;15(5):e0229889. doi: 10.1371/journal.pone.0229889 (PMC7217464; doi:10.1371/journal.pone.0229889)
Supplement: S1 File — (DOCX) [file pone.0229889.s001.docx]

**Instigation of indigenous thermophilic bacterial consortia for enhanced oil recovery from high temperature oil reservoirs**

**Neha Sharma^1^, Meeta Lavania^1,*^, Vipin Kukreti^2^, Banwari Lal^1^**

**^1^ Microbial Biotechnology Division, The Energy & Resources Institute, Darbari Seth Block, IHC Complex, New Delhi, India- 110003**

**^2^** Institute of Reservoir Studies Oil and Natural Gas Corporation Limited, Chandkeda Campus Ahmedabad, Gujarat

**^*^ Corresponding author**

**E-mail:meetal@teri.res.in**

**Supporting information**

**Experiment 1:** **Evaluation of biosurfactant through oil spreading assay**

**Methodology**

The oil spreading method was used to quantify the amount of biosurfactant present in the crude cell free sample. A petri dish was filled with 30 ml of deionized water and 30 µl of crude oil was added on the water which forms oil film. 10µl of culture surfactant was poured on the surface of the oil film that serves as treated. 10 µl of deionized water was added on the surface of oil film which serves as control.

**Result**

The activity of biosurfactant was monitored as the diameter of clear zone was formed on the oil film (Fig. S1). It was clearly evident that the biosurfactant produced by TERIK was effective and can be capable of reducing interfacial tension between the oil-water and oil-rock.

**Discussion**

The presence of biosurfactant in the cell free extract can be monitored through the displacement of oil and formation of clearing zone. The diameter of the clearing zone on the oil surface correlates to surfactant activity also known as oil displacement activity. The oil spreading method is rapid and easy method.


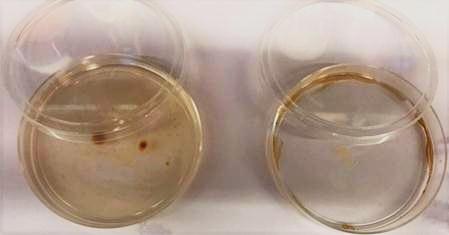


Treated

Control

**S1Fig.** Oil spreading assay represents clearing zone due to the activity of biosurfactant

**Experiment 2: Estimation of biosurfactant through drop collapse method**

**Methodology**

In drop collapse method, two drops of crude oil was placed on tape and then on one drop water was placed (control) and on to other drop biosurfactant was placed (serves as treated). The change in drop size was monitored.

**Result**

The drop collapse assay is rapid, easy and requires small volume of sample. This assay has been applied several times for screening purposes. The drops of a cell suspension or of culture supernatant are placed on an oil coated surface. The drop of biosurfactant spread or even collapse because of the force or interfacial tension between the liquid drop and the hydrophobic surface was reduced. The polar water molecules are repelled from the hydrophobic surface and the drops remain stable (Fig. S2). The stability of drop depends on the surfactant concentration.


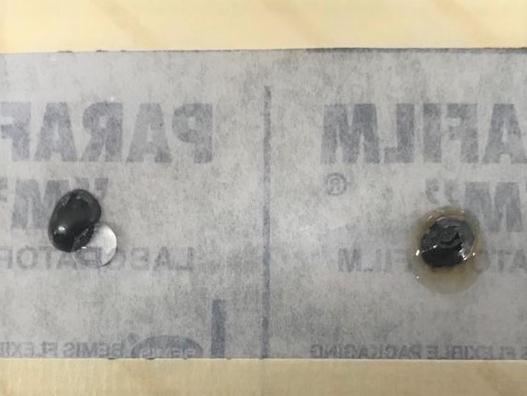


Control

Treated

**Fig. S2** Drop collapse assay: biosurfactant drop was collapsed

**Experiment 3:** **Detection of carbohydrate through molisch’s test**

**Methodology**

To detect the presence of carbohydrates in the biosurfactant, the solution was first treated with a strong acid that hydrolyze the carbohydrate to monosaccharide. A byproduct furfurals was formed which reacted with alpha-naphthol of Molisch’s reagent that results into purple ring. The positive control was glucose solution and sample (biosurfactant) was monitored for carbohydrates

**Result**

The results were clearly evident in Fig. S3 that represents purple ring in the glucose and biosurfactant sample isolated from TERIK. The presence of purple ring shows the presence of byproduct furfural formed during the hydrolysis of carbohydrate.


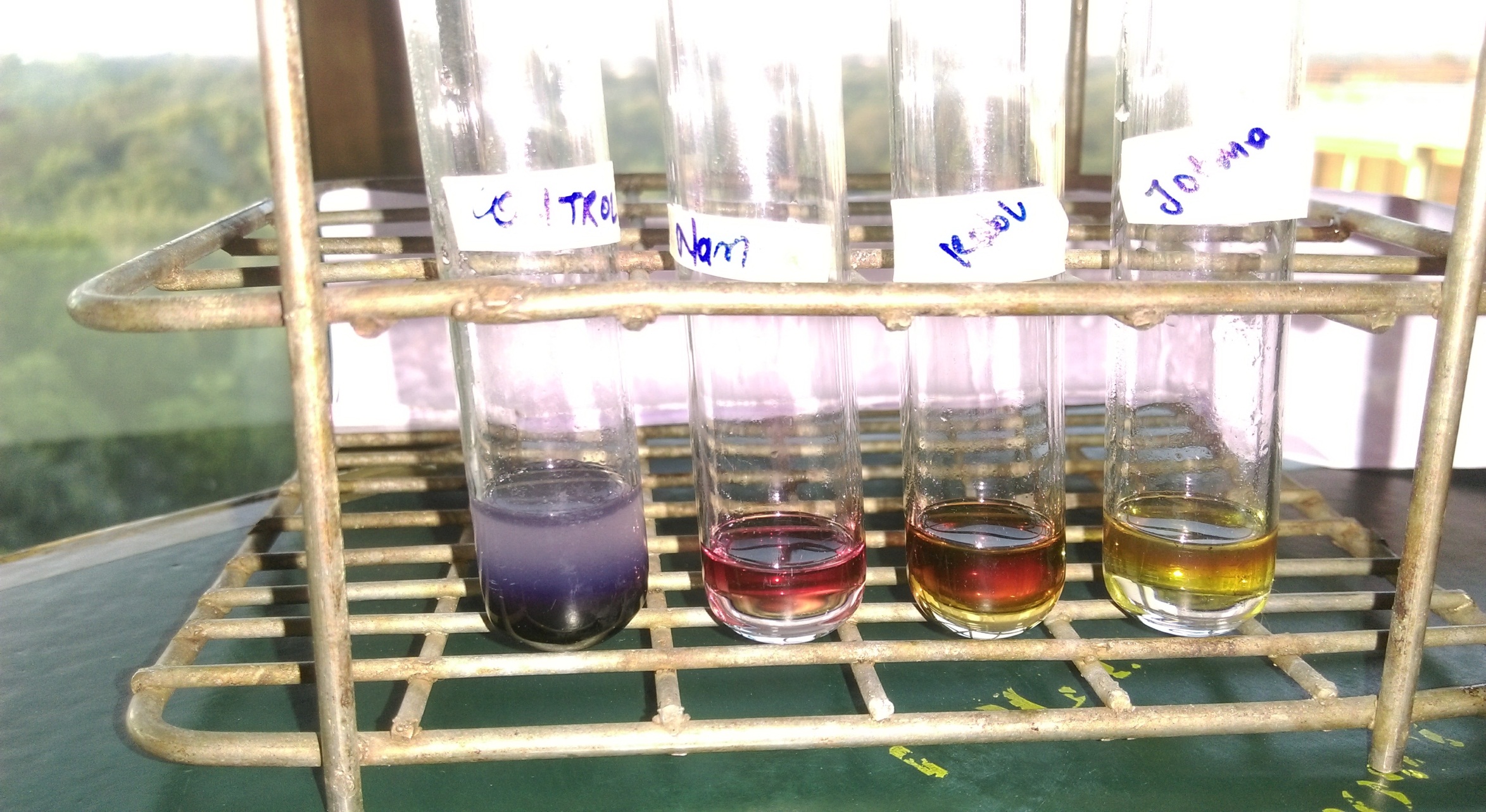


Positive Control

Sample

**Fig.S3** Qualitative test (Molisch’s Test) of biosurfactant

**References:**

Batista, S., Mounteer, A., Amorim, F., et al. 2006.Isolation and characterization of biosurfactant/bioemulsifier producing bacteria from petroleum contaminated sites. Bioresour Technol. 97(6), 868–875.

Bodour, A., Drees, K., Maier, R.,2003. Distribution of biosurfactant-producing bacteria in undisturbed and contaminated arid southwestern soils. Appl Environ Microbiol. 69(6),3280–3287.

Maczek, J., Junne, S., Gotz, P., 2007. Examining biosurfactant producing bacteria—an example for an automated search for natural compounds. Application Note CyBio AG.

Morikawa, M., Hirata, Y., Imanaka, T., 2000. A study on the structure-function relationship of lipopeptide biosurfactants. Biochim Biophys Acta.1488(3), 211–218.

Plaza, G., Zjawiony, I., Banat, I., 2006. Use of different methods for detection of thermophilic biosurfactant-producing bacteria from hydrocarbon-contaminated bioremediated soils. J Petro Science Eng. 50(1),71–77.

Youssef, N., Duncan, K., Nagle, D. et al. 2004. Comparison of methods to detect biosurfactant production by diverse microorganisms. J Microbiol Methods. 56(3), 339–347.
