# Supplementary figures and images for: Instigation of indigenous thermophilic bacterial consortia for enhanced oil recovery from high temperature oil reservoirs
Source: PLoS One. 2020 May 12;15(5):e0229889. doi: 10.1371/journal.pone.0229889 (PMC7217464; doi:10.1371/journal.pone.0229889)

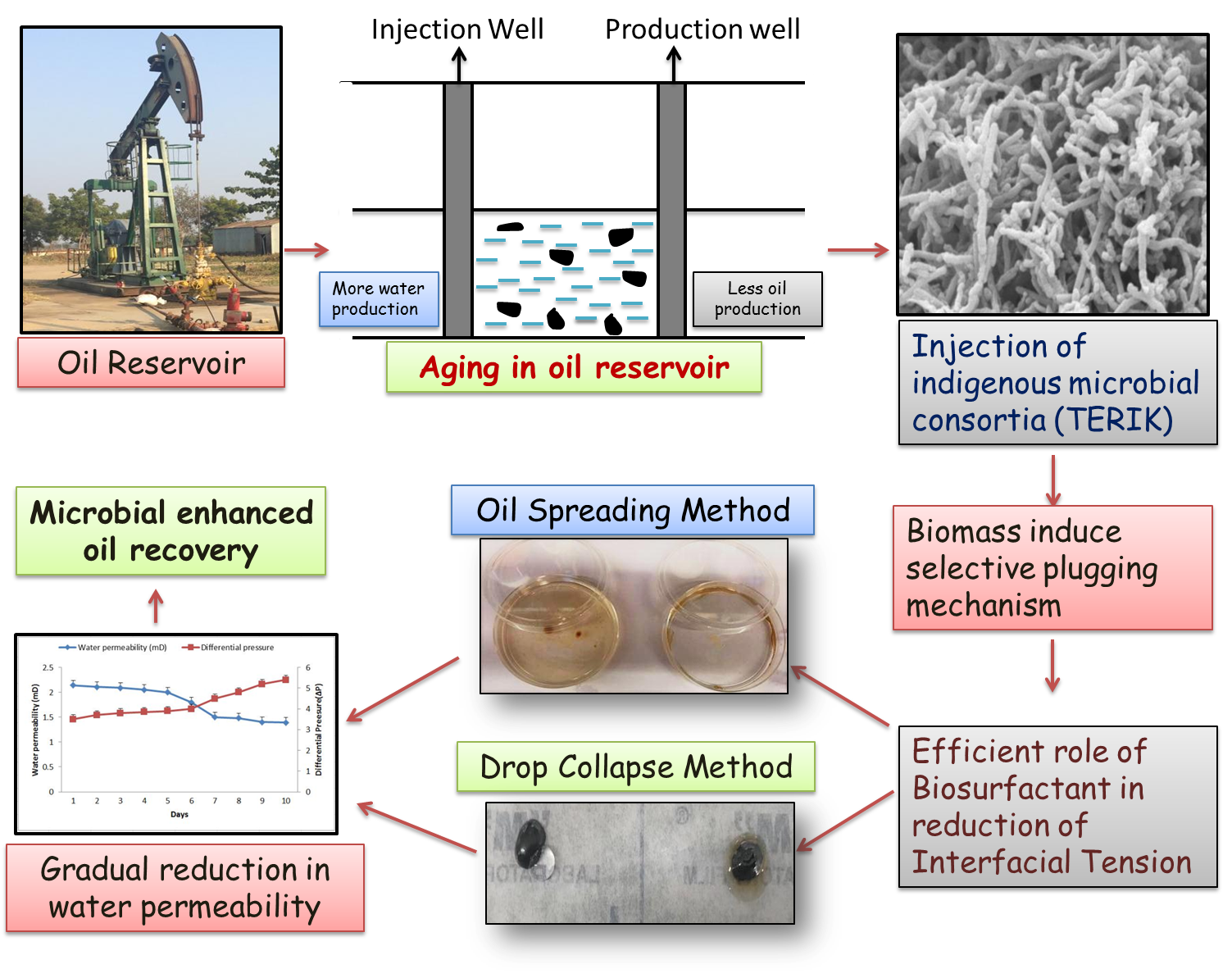

Supplement: S1 Graphical abstract — (PNG) [file pone.0229889.s002.png]
